# Deucravacitinib, an oral, selective, allosteric tyrosine kinase 2 inhibitor, in Japanese patients with moderate to severe plaque, erythrodermic, or generalized pustular psoriasis: Efficacy and safety results from an open‐label, phase 3 trial

**DOI:** 10.1111/1346-8138.17074

**Published:** 2024-01-24

**Authors:** Shinichi Imafuku, Yukari Okubo, Yayoi Tada, Mamitaro Ohtsuki, Elizabeth Colston, Andrew Napoli, Yanqiu Shao, Subhashis Banerjee, Akimichi Morita

**Affiliations:** ^1^ Department of Dermatology Fukuoka University Faculty of Medicine Fukuoka Japan; ^2^ Department of Dermatology Tokyo Medical University Tokyo Japan; ^3^ Department of Dermatology Teikyo University School of Medicine Tokyo Japan; ^4^ Department of Dermatology Jichi Medical University Tochigi Japan; ^5^ Bristol Myers Squibb Princeton New Jersey USA; ^6^ Department of Geriatrics and Environmental Dermatology Nagoya City University Graduate School of Medical Sciences Nagoya Japan

**Keywords:** Asian population, clinical trial, phase 3, psoriasis, tyrosine kinase 2

## Abstract

Deucravacitinib, an oral, selective, allosteric tyrosine kinase 2 inhibitor, is approved in Japan for adult patients with plaque (PP), generalized pustular (GPP), and erythrodermic (EP) psoriasis who have had an inadequate response to conventional systemic therapies. This approval is based on results from the global phase 3 POETYK PSO‐1 and PSO‐2 trials in which deucravacitinib was associated with significantly improved efficacy outcomes compared with placebo in adults with moderate to severe plaque psoriasis, and results described here from POETYK PSO‐4, an open‐label, single‐arm, phase 3 trial (NCT03924427), which evaluated the efficacy and safety of deucravacitinib 6 mg once daily in adult Japanese patients with PP, GPP, or EP. The coprimary endpoints were the proportion of patients achieving a ≥75% reduction from baseline in the Psoriasis Area and Severity Index (PASI 75) and a static Physician's Global Assessment score of 0 (clear) or 1 (almost clear) (sPGA 0/1) with at least a two‐point improvement from baseline at week 16. Nonresponder imputation was used for missing data. Efficacy responses, adverse events (AEs), and serious AEs (SAEs) were recorded for up to 52 weeks. Seventy‐four patients were treated (PP, *n* = 63; GPP, *n* = 3; EP, *n* = 8). At week 16, 76.2%, 66.7%, and 37.5% of patients with PP, GPP, and EP, respectively, had achieved PASI 75, and 82.5%, 0.0%, and 50.0% had achieved sPGA 0/1. Responses were overall maintained through week 52. AEs occurred in 74.6% of patients with PP, 100% of patients with GPP, and 87.5% of patients with EP. The most common AEs were nasopharyngitis and acne. Rates of SAEs and discontinuations were low. There were no deaths. Deucravacitinib was effective and well tolerated in Japanese patients with moderate to severe PP and in a limited number of patients with GPP or EP.

## 1 INTRODUCTION

Psoriasis is a chronic inflammatory disease that generally requires lifelong treatment and management.^1^, ^2^ In Japan, the prevalence rate of psoriasis is 0.3%.^3^ Plaque psoriasis (PP) is the most common subtype, with an estimated prevalence of 97.4% of Japanese psoriasis cases.^3^ The national prevalence of generalized pustular psoriasis (GPP) and erythrodermic psoriasis (EP) among psoriasis patients in Japan has been estimated at 1.1% and 0.4%, respectively.^3^ Among the systemic therapeutic options that have been approved for moderate to severe PP in Japan, oral medications such as apremilast appear to be the most frequently used, followed by biologic treatments.^1^, ^4^ Biologic therapies, however, are only delivered through injection or infusion, can lose efficacy over time, and are regulated based on guidance by the Japanese Dermatological Association (JDA).^2^, ^5^ GPP and EP are managed with cyclosporine, retinoids, and other agents that may have significant side effects.^6^, ^7^, ^8^ Novel, oral, targeted treatments that are convenient, safe, and efficacious are needed.

Tyrosine kinase 2 (TYK2) is an intracellular mediator of cytokine signaling (e.g., interleukin [IL]‐23 and type I interferons) essential to psoriasis pathogenesis.^9^, ^10^, ^11^ Genetic loss of TYK2 function has been associated with a lower risk of developing immune‐mediated diseases, including psoriasis, without increased safety risks.^9^, ^10^, ^11^, ^12^, ^13^ Deucravacitinib is an oral, selective, allosteric TYK2 inhibitor that uniquely inhibits TYK2 by binding to the regulatory domain rather than to the more conserved catalytic domain where Janus kinase (JAK) 1/2/3 inhibitors bind, thus avoiding the off‐target effects found with JAK inhibitors.^9^, ^14^, ^15^, ^16^ It is ≥100‐fold more selective for TYK2 than JAK 1/3 and ≥2000‐fold more selective for TYK2 than JAK 2 in cells.^9^, ^17^ Deucravacitinib is approved in Japan for patients with PP, GPP, and EP who have had an inadequate response to conventional systemic therapies; it is also approved by the USA and other countries for the treatment of adults with moderate‐to‐severe PP who are candidates for systemic therapy or phototherapy.^18^, ^19^

Findings from the global, phase 3, double‐blind POETYK PSO‐1 and PSO‐2 trials in patients with moderate to severe PP showed that deucravacitinib was significantly more effective than placebo and apremilast based on the coprimary endpoints of a ≥75% reduction in the Psoriasis Area and Severity Index (PASI 75) and a static Physician's Global Assessment score of 0 (clear) or almost clear (1) with at least a two‐point improvement from baseline (sPGA 0/1) at week 16 and was well tolerated.^20^, ^21^ Clinical responses were maintained through 52 weeks in patients who received continuous deucravacitinib treatment and were improved in patients who crossed over from placebo to deucravacitinib at week 16 in these trials. A subgroup analysis of the Japanese patients in the POETYK PSO‐1 trial determined that the efficacy and safety of deucravacitinib in this group was consistent with that of the global trial population.^22^

This report presents data from the single‐arm, open‐label, phase 3 POETYK PSO‐4 trial, which assessed the efficacy and safety of deucravacitinib over 52 weeks in Japanese patients with moderate to severe PP, GPP, or EP.

## 2 METHODS

### 2.1 Study design

**Figure 1:**
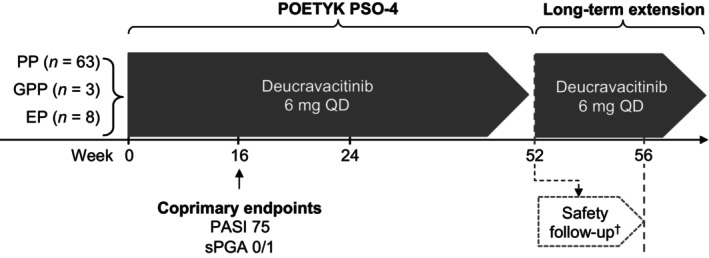
The POETYK PSO‐4 study design. ^†^Patients who did not roll over into the POETYK LTE trial entered a 4‐week safety follow‐up through week 56. EP, erythrodermic psoriasis; GPP, generalized pustular psoriasis; PASI 75, ≥75% reduction from baseline in the Psoriasis Area and Severity Index; PP, plaque psoriasis; QD, once daily; sPGA 0/1, static Physician's Global Assessment score of 0 (clear) or 1 (almost clear) with at least a two‐point improvement from baseline.

POETYK PSO‐4 (NCT03924427) was a phase 3, multicenter, single‐arm, open‐label trial conducted at 25 sites in Japan. This 52‐week trial consisted of three periods (Figure 1). The screening period lasted up to 4 weeks. Patients with GPP and EP began enrolling at the start of the trial and continued throughout; patients with PP began enrolling after 52 Japanese patients had enrolled in POETYK PSO‐1. The treatment period lasted 52 weeks. Patients completing 52 weeks of treatment could enroll in the open‐label, POETYK long‐term extension (LTE) trial; patients who did not enroll in the LTE were monitored for a 4‐week follow‐up period.

This study was conducted in accordance with Good Clinical Practice, as defined by the International Council for Harmonization and the Declaration of Helsinki. Institutional review board approval was received at each site and all patients provided written informed consent.

### 2.2 Study population

Japanese adults ≥20 years of age with moderate to severe psoriasis who were considered by the investigator to be candidates for phototherapy or systemic therapy were enrolled. For PP, moderate to severe psoriasis was defined as stable (no morphology changes or significant flares of disease activity in the previous 6 months in the opinion of investigator), with PASI ≥12, sPGA ≥3, and BSA involvement ≥10% at screening and day 1. For GPP, moderate to severe psoriasis was defined as a history of or newly diagnosed disease, a stable treatment regimen for ≥2 weeks before day 1, erythematous lesion pustules with ≥10% BSA involvement and a score of ≥2 on skin lesions at screening and day 1, and a total JDA severity index score <14 at screening; for EP, moderate to severe psoriasis was defined as a history of or newly diagnosed disease, ≥80% BSA involvement at screening and day 1, and a history of plaque psoriasis. Full inclusion and exclusion criteria can be found in Table 1.

**Table 1:** Inclusion and exclusion criteria.

Inclusion criteria
**Patient population**
1. Men and women aged ≥20 years at the time of screening visit
**For patients with plaque psoriasis**
1. Men and women diagnosed with stable plaque psoriasis for 6 months or more; stable plaque psoriasis is defined as no morphology changes or significant flares of disease activity in the opinion of the investigator
2. Deemed by the investigator to be a candidate for phototherapy or systemic therapy
3. ≥10% of body surface area involvement at screening visit and day 1
4. PASI score ≥12 and sPGA score ≥3 at screening visit and day 1
**For patients with generalized pustular psoriasis**
1. Men and women with a history of or newly diagnosed generalized pustular psoriasis based on Japanese Dermatological Association criteria
2. Have generalized pustular psoriasis and have been on a stable treatment regimen for at least 2 weeks prior to day 1
3. At screening visit and day 1, have erythematous lesions with pustules involving ≥10% of body surface area (i.e., score of ≥2 on this skin symptom)
4. At screening visit, total Japanese Dermatological Association severity index score <14
5. Deemed by the investigator to be a candidate for phototherapy or systemic therapy
**For patients with erythrodermic psoriasis**
1. Men and women with a prior history of or newly diagnosed erythrodermic psoriasis
2. A history of plaque‐type psoriasis
3. At screening visit and day 1, have ≥80% of body surface area involvement
4. Deemed by the investigator to be a candidate for phototherapy or systemic therapy
**For women**
1. WOCBP must have a negative serum pregnancy test at screening visit, and a negative urine pregnancy test (minimum sensitivity 25 IU/L or equivalent units of hCG) within 24 h prior to the start of study drug
2. Women must not be pregnant, lactating, breastfeeding, or planning pregnancy during the study period; women must not enroll even if lactation or breastfeeding is interrupted
3. WOCBP must agree to use correctly a highly effective method(s) of contraception for the duration of treatment (52 weeks) with study drug(s) BMS‐986165 plus 5 half‐lives of study drug (3 days) plus 30 days (duration of ovulatory cycle) for a total of 33 days posttreatment completion (total of 33 days after last dose of study drug); WOCBP who are continuously not heterosexually active are exempt from contraceptive requirements, but must still undergo pregnancy testing as described in this protocol
**For men**
1. Male patients who are sexually active with WOCBP must agree to follow instructions for method(s) of contraception for the duration of treatment with study treatment(s) plus 5 half‐lives of the study treatment (3 days) for a total of 3 days posttreatment completion; in addition, male patients must be willing to refrain from sperm donation during this time

Exclusion criteria
1. Patients with guttate, inverse, or drug‐induced psoriasis at screening or day 1 *Note*: The study allows men and women diagnosed with generalized pustular psoriasis or erythrodermic psoriasis
2. History or evidence of outpatient active infection and/or febrile illness within 7 days of day 1
3. Any untreated bacterial infection within 60 days prior to day 1
4. Any ongoing evidence of chronic, bacterial infection (e.g., chronic pyelonephritis, chronic osteomyelitis, chronic bronchiectasis)
5. Any history of proven infection of a joint prosthesis in which the prosthesis was not removed or replaced, or received antibiotics for suspected infection of a joint prosthesis in which the prosthesis was not removed or replaced
6. Received live vaccines within 60 days prior to day 1, or plans to receive a live vaccine during the study, or within 60 days after completing study treatment
7. Presence of herpes zoster lesions at screening or day 1
8. History of serious herpes zoster or serious herpes simplex infection which includes, but is not limited to, any episode of disseminated herpes simplex, multidermatomal herpes zoster, herpes encephalitis, ophthalmic herpes, or recurrent herpes zoster (recurrent is defined as 2 episodes within 2 years)
9. Evidence of, or test positive for, hepatitis B virus (HBV) at screening; positive hepatitis B laboratory testing is defined as: (1) HBsAg+ OR (2) presence of HBV DNA OR (3) positive hepatitis B core antibody patients with detectable HBV DNA by PCR OR (4) HBsAg+ patients with detectable HBV DNA by PCR; patients with HBsAg− but anti‐HBsAb+, and/or anti‐HBcAb+ must also be tested for quantitative HBV DNA; patients with HBV DNA ≥2.1 log copy/mL (20 IU/mL) are excluded from the study; patients with HBV DNA <2.1 log copy/mL (20 IU/mL) can be enrolled in the study but must undergo periodic monitoring of HBV DNA and liver enzymes, such as AST and ALT; monitoring should be performed every 4 weeks throughout the duration of the study and at the safety follow‐up visit at week 56
10. Evidence of, or test positive for, HCV at screening; a positive test for HCV is defined as: (1) positive for hepatitis C antibody (anti‐HCV Ab) and (2) positive via a confirmatory test for HCV (e.g., HCV PCR)
11. Positive for HIV by antibody testing (HIV‐1 and −2 Ab) at screening
12. Any history of known or suspected congenital or acquired immunodeficiency state or condition that would compromise the patient's immune status (e.g., history of opportunistic infections [*Pneumocystis jirovecii pneumonia*, histoplasmosis, or coccidioidomycosis], history of splenectomy, primary immunodeficiency)
13. History of active tuberculosis (TB) prior to screening visit, regardless of completion of adequate treatment
14. Signs or symptoms of active TB (e.g., fever, cough, night sweats, weight loss) during screening as judged by the investigator
15. Any imaging of the chest (e.g., chest x‐ray, chest computed tomography scan) obtained during the screening period or anytime within 6 months prior to screening with documentation, showing evidence of current active or history of active pulmonary TB
16. LTBI defined as positive interferon gamma release assay (IGRA), by QuantiFERON®‐TB Gold testing at screening, in the absence of clinical manifestations *Note*: A patient is eligible if (1) there are no current signs or symptoms of active TB AND (2) the patient has received adequate documented treatment for LTBI within 5 years of screening OR has initiated prophylactic treatment for LTBI per local guidelines and is rescreened after 1 month of treatment. To continue in the study, the patient must agree to complete a locally recommended course of treatment for LTBI *Note*: A patient with an IGRA test that is indeterminate with no signs or symptoms of active TB must be retested for confirmation. If the second test is again indeterminate, the patient will be excluded from the study. If the retest result is positive, the patient should be treated as having LTBI. If the retest result is negative, the patient may be eligible provided no other exclusion criteria for TB are met
17. Any major surgery within 8 weeks prior to day 1, or any planned surgery for the first 52 weeks of the study
18. Has donated blood >500 mL within 4 weeks prior to day 1, or plans to donate blood during the course of the study
19. Drug or alcohol abuse, as determined by the investigator, within 6 months prior to day 1
20. Medical marijuana or prescription marijuana taken for medicinal reasons
21. Any major illness/condition or evidence of an unstable clinical condition (e.g., renal, hepatic, hematologic, gastrointestinal, endocrine, pulmonary, psychiatric, neurologic, immunologic, local active infection/infectious illness) that, in the investigator's judgment or after consultation with the Medical Monitor, will substantially increase the risk to the patient if he or she participates in the study
22. Unstable cardiovascular disease, defined as a recent clinical cardiovascular event (e.g., unstable angina, myocardial infarction, stroke, rapid atrial fibrillation) in the last 3 months before screening, or a cardiac hospitalization (e.g., revascularization procedure, pacemaker implantation) within 3 months before screening
23. Has uncontrolled arterial hypertension characterized by a systolic blood pressure >160 mm Hg or diastolic blood pressure >100 mm Hg *Note*: Determined by two consecutive elevated readings. If an initial BP reading exceeds this limit, the BP may be repeated once after the patient has rested sitting for ≥10 min. If the repeat value is less than the criterion limits, the second value may be accepted
24. Class III or IV congestive heart failure by New York Heart Association criteria
25. Has cancer or history of cancer (solid organ or hematologic including myelodysplastic syndrome) or lymphoproliferative disease within the previous 5 years (other than resected cutaneous basal cell or squamous cell carcinoma, or carcinoma of cervix in situ that has been treated with no evidence of recurrence)
26. Any uncontrolled neuropsychiatric illness judged as clinically significant by the investigator during screening or at day 1 OR any lifetime history of suicidal ideation, suicidal behavior, or suicidal attempts by medical history or by eC‐27 SSRS documentation, or by answering “yes” to Question 4 or 5 for suicidal ideation on the eC‐SSRS at screening or at day 1, or is clinically deemed to have a suicide risk by the investigator
27. Prior exposure to investigational product
28. If the patient has received biologics previously, the following exclusion criteria for washout will apply: Antibodies to IL‐12, IL‐17, or IL‐23 (e.g., ustekinumab, secukinumab, tildrakizumab, ixekizumab, guselkumab) within 6 months of day 1 TNF inhibitor(s) (e.g., etanercept, adalimumab, infliximab, certolizumab) within 2 months of day 1 Agents that modulate integrin pathways to impact lymphocyte trafficking (e.g., natalizumab), or agents that modulate B cells or T cells (e.g., alemtuzumab, abatacept, or visilizumab) within 3 months of day 1 Rituximab within 6 months of day 1
29. Has received systemic nonbiologic psoriasis medications and/or any systemic immunosuppressants (including, but not limited to, methotrexate, azathioprine, cyclosporine, JAK inhibitors, 6‐thioguanine, mercaptopurine, mycophenolate mofetil, hydroxyurea, tacrolimus, oral or injectable corticosteroids, retinoids, 1,25‐dihydroxy vitamin D3 and analogs, psoralens, sulfasalazine, or fumaric acid derivatives) within 4 weeks prior to day 1 with the following exceptions: For patients with erythrodermic psoriasis or generalized pustular psoriasis, use of prednisone or its equivalent at a daily dose ≤10 mg/day is permitted For patients with generalized pustular psoriasis, one of the following agents is permitted: cyclosporine, methotrexate, or retinoids For patients with erythrodermic psoriasis, concomitant use of cyclosporine is permitted until week 2
30. Has used leflunomide within 6 months prior to day 1
31. Has used opioid analgesics within 4 weeks prior to day 1
32. Has received lithium, antimalarials, or intramuscular gold within 4 weeks of the first administration of any study medication
33. Has received phototherapy (including either oral and topical PUVA light therapy, ultraviolet B, or self‐treatment with tanning beds or therapeutic sunbathing) within 4 weeks prior to day 1
34. Has used topical medications/treatments that could affect psoriasis evaluation (including, but not limited to, ultra‐high to moderate potency corticosteroids [WHO Classes I–V], >3% salicylic acid, urea, alpha‐ or beta‐hydroxyl acids, anthralin, calcipotriene, topical vitamin D derivatives, retinoids, tazarotene, methoxsalen, trimethylpsoralens, picrolimus, and tacrolimus) within 2 weeks prior to day 1 *Note*: Low potency topical steroids (WHO Classes VI and VII) are permitted on the palms, soles, face, and intertriginous areas as well as bland emollients (without urea or alpha or beta hydroxy acids). They should not be used within 24 h before any study visit.
35. Use of shampoos that contain corticosteroids, coal tar, >3% salicylic acid, or vitamin D3 analogues within 2 weeks before day 1
36. Has received an experimental antibody or experimental biologic therapy within the previous 6 months, OR received any other experimental therapy or new investigational agent within 30 days or 5 half‐lives (whichever is longer) before day 1 OR is currently enrolled in an investigational study
37. The following conditions for patients with generalized pustular psoriasis: Drug‐induced generalized pustular psoriasis Subcorneal pustular dermatosis Previous psoriasis vulgaris who experienced temporary pustulation Circinate annular form of generalized pustular psoriasis
38. For patients with psoriatic arthritis: Received any parenteral corticosteroid injections within 6 weeks of screening
39. Any other sound medical, psychiatric, and/or social reason as determined by the investigator Received any parenteral corticosteroid injections within 6 weeks of screening
40. At screening Absolute white blood cell count <3000/mm^3^ Absolute lymphocyte count <500/mm^3^ Absolute neutrophil count <1000/mm^3^ Platelet count <100 000/mm^3^ Hemoglobin <9 g/dL ALT and/or AST >3× upper limit of normal Total, unconjugated and/or conjugated bilirubin >2× upper limit of normal Thyroid‐stimulating hormone outside the normal reference range AND free T4 (thyroxine) or T3 (triiodothyronine) outside the normal reference range
41. Electrocardiogram abnormalities that are considered clinically significant and would pose an unacceptable risk to the patient if participating in the study
42. Renal impairment based on an estimated glomerular filtration rate <45 mL/min
43. Inability to be venipunctured and/or tolerate venous access
44. Any other significant laboratory abnormalities that, in the opinion of the investigator, might place the patient at unacceptable risk for participation in this study
45. (1→3)‐beta‐D‐glucan positive
46. History of any significant drug allergy (such as anaphylaxis)
47. Prisoners or patients who are involuntarily incarcerated (Note: under certain specific circumstances a person who has been imprisoned may be included or permitted to continue as a patient; strict conditions apply and Bristol Myers Squibb approval is required)
48. Patients who are compulsorily detained for treatment of either a psychiatric or physical (e.g., infectious disease) illness
49. Inability to comply with restrictions and prohibited activities/treatments as listed in the study protocol
50. Site personnel or their immediate family

Abbreviations: ALT, alanine aminotransferase; AST, aspartate aminotransferase; eC‐SSRS, electronic Columbia‐Suicide Severity Rating Scale; HBsAg−, negative hepatitis B surface antigen; HBsAg+, positive hepatitis B surface antigen; HBV, hepatitis B virus; hCG, human chorionic gonadotropin; HCV, hepatitis C virus; HIV, human immunodeficiency virus; IL, interleukin; JAK, Janus kinase; LTBI, latent tuberculosis infection; PASI, Psoriasis Area and Severity Index; PUVA, psoralen ultraviolet light‐A; sPGA, static Physician’s Global Assessment; TNF, tumor necrosis factor; WHO, World Health Organization; WOCBP, women of childbearing potential.

### 2.3 Treatment protocol

Eligible patients received open‐label treatment with deucravacitinib 6 mg once daily for 52 weeks. Efficacy and safety assessments were performed at baseline, weeks 1, 2, 4, 8, 12, and 16, then every 4 weeks through week 52.

### 2.4 Outcome measures

The coprimary endpoints were the proportion of patients who achieved PASI 75 and sPGA 0/1 at week 16. Secondary efficacy endpoints over 52 weeks included PASI 75, sPGA 0/1, ≥90% and 100% reductions from baseline in PASI (PASI 90 and PASI 100), sPGA 0 (clear), scalp‐specific Physician's Global Assessment of 0 (clear) or 1 (almost clear) (ss‐PGA 0/1) and change from baseline in the Psoriasis Scalp Severity Index (PSSI) in patients with a baseline ss‐PGA score ≥3 (moderate to severe), Physician's Global Assessment–Fingernail of 0 (clear) or 1 (almost clear) (PGA‐F 0/1) and percentage change from baseline in the modified Nail Psoriasis Severity Index (mNAPSI) in patients with a baseline PGA‐F score ≥3 (moderate to severe), and palmoplantar Physician's Global Assessment score of 0 (clear) or 1 (almost clear) (pp‐PGA 0/1) and percentage change from baseline in the palmoplantar Psoriasis Area and Severity Index (pp‐PASI) in patients with a baseline pp‐PGA score ≥3 (moderate to severe).

### 2.5 Safety measures

Adverse events (AEs), serious AEs (SAEs), and AEs leading to discontinuation were recorded over 52 weeks. AEs of interest were based on known psoriasis comorbidities and safety issues associated with current marketed immunomodulatory compounds. These included certain skin‐related AEs (acne, folliculitis), infection‐related AEs (influenza, herpes zoster, opportunistic infections, and tuberculosis), creatine kinase elevation, and malignancy. Adjudicated AEs included select infections, cardiovascular events, and suicidal ideation and behavior. These events were adjudicated by independent, external, blinded, subspecialty expert adjudicators. For events characterized as AEs of interest or adjudicated AEs, prespecified Medical Dictionary for Regulatory Activities preferred terms were used to identify events. Hematologic, lipid, and chemistry laboratory parameters were evaluated throughout the trial.

### 2.6 Statistical methods

Sample size was based on including an adequate number of Japanese patients to evaluate the safety of deucravacitinib. Assuming a combined 80 patients with PP, GPP, or EP, there was a 90% chance of observing an AE that occurred in at least 3% of the population. A sample size of 60 patients with PP with an assumed 60% PASI 75 response rate was predicted to provide a 95% confidence interval width of approximately 25% for the PASI 75 response rate.^23^

As this was a single‐arm study, no statistical tests for treatment comparisons were conducted. Categorical data were summarized as frequency counts and percentages. Continuous endpoints were summarized with descriptive statistics. Efficacy and safety analyses were conducted using the as‐treated population (all enrolled patients who received at least one dose of study treatment). Nonresponder imputation (NRI) was the primary method of imputation for the coprimary endpoints, PASI 75 and sPGA 0/1, for patients who discontinued treatment prior to week 16 or had missing week 16 data. Imputed results of these endpoints by NRI were also available through week 52. Safety results are presented for weeks 0–52 by frequencies and exposure‐adjusted incidence rates (EAIRs) per 100 person‐years (PY). Laboratory analyses were summarized as raw values, as change from baseline, and by postbaseline maximum value.

## 3 RESULTS

### 3.1 Patient characteristics

Between April 10, 2019 and March 24, 2021, 83 patients were enrolled in the trial. Of these, there were seven screen failures, one patient withdrawal, and one SAE (worsening EP) prior to randomization, leaving 74 patients who received treatment and were included in the as‐treated population (PP, *N* = 63; GPP, *N* = 3; EP, *N* = 8); 68 completed treatment. Baseline patient demographics and disease characteristics were balanced across the three groups (Table 2).

**Table 2:** Baseline patient demographics and disease characteristics.

Parameter	Plaque psoriasis (*n* = 63)	Generalized pustular psoriasis (*n* = 3)	Erythrodermic psoriasis (*n* = 8)
Age, mean (SD), years	49.1 (12.1)	43.3 (17.6)	46.5 (5.4)
Weight, mean (SD), kg	69.5 (14.2)	72.4 (7.0)	78.8 (16.0)
BMI, mean (SD), kg/m^2^	24.9 (4.4)	27.7 (5.0)	26.5 (5.1)
Sex, *n* (%)			
Female	15 (23.8)	2 (66.7)	0
Male	48 (76.2)	1 (33.3)	8 (100)
Disease duration, mean (SD), year	15.4 (10.7)	10.0 (12.7)	18.6 (5.5)
Prior systemic treatment, *n* (%)			
Biologic	10 (15.9)	1 (33.3)	2 (25.0)
Nonbiologic	35 (55.6)	2 (66.7)	4 (50.0)
No prior systemic therapy	18 (28.6)	0	2 (25.0)
PASI, mean (SD)	21.1 (9.2)	20.8 (12.3)	44.9 (10.6)
sPGA, *n* (%)			
0 (clear)	0	0	0
1 (almost clear)	0	1 (33.3)	0
2 (mild)	0	0	0
3 (moderate)	56 (88.9)	2 (66.7)	7 (87.5)
4 (severe)	7 (11.1)	0	1 (12.5)
BSA involvement, mean (SD), %	30.3 (18.6)	37.0 (15.4)	86.8 (6.2)
PSSD symptom score, mean (SD)	39.8 (23.6)	46.6 (4.0)	35.7 (26.0)
DLQI, mean (SD)	9.1 (4.5)	9.7 (6.4)	7.5 (5.6)
ss‐PGA ≥3, *n* (%)	35 (55.6)	2 (66.7)	5 (62.5)
PSSI score, mean (SD)^a^	32.4 (14.9)	36.5 (12.0)	45.4 (12.6)
PGA‐F ≥ 3, *n* (%)	10 (15.9)	0	4 (50)
mNAPSI score, mean (SD)^b^	29.7 (12.3)	0	50.5 (19.8)
pp‐PGA ≥3, *n* (%)	4 (6.3)	0	1 (12.5)
pp‐PASI, mean (SD)^c^	24.0 (22.2)	0	21.6 (−)

Abbreviations: BMI, body mass index; BSA, body surface area; DLQI, Dermatology Life Quality Index; mNAPSI, modified Nail Psoriasis Severity Index; PASI, Psoriasis Area and Severity Index; PGA‐F, Physician's Global Assessment–Fingernail; pp‐PASI, palmoplantar Psoriasis Area and Severity Index; pp‐PGA, palmoplantar Physician's Global Assessment; PSSD, Psoriasis Symptoms and Signs Diary; PSSI, Psoriasis Scalp Severity Index; SD, standard deviation; sPGA, static Physician's Global Assessment; ss‐PGA, scalp‐specific Physician's Global Assessment.

^a^ In patients with baseline ss‐PGA score ≥3.

^b^ In patients with baseline PGA‐F score ≥3.

^c^ In patients with baseline pp‐PGA score ≥3.

### 3.2 Efficacy

#### 3.2.1 Plaque psoriasis

**Figure 2:**
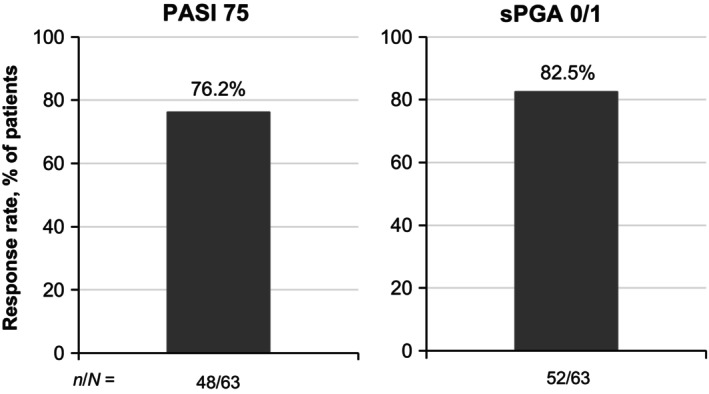
PASI 75 and sPGA 0/1 responses at week 16 in patients with PP (NRI). NRI, nonresponder imputation; PASI 75, ≥75% improvement from baseline in the Psoriasis Area and Severity Index; PP, plaque psoriasis; sPGA 0/1, static Physician's Global Assessment score of 0 (clear) or 1 (almost clear) with at least a two‐point improvement from baseline.

**Figure 3:**
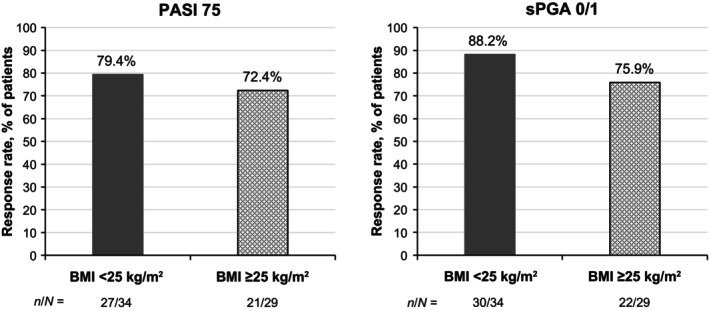
PASI 75 and sPGA 0/1 at week 16 in patients with PP by baseline BMI (NRI). BMI, body mass index; NRI, nonresponder imputation; PASI 75, ≥75% improvement from baseline in the Psoriasis Area and Severity Index; PP, plaque psoriasis; sPGA 0/1, static Physician's Global Assessment score of 0 (clear) or 1 (almost clear) with at least a two‐point improvement from baseline.

**Figure 4:**
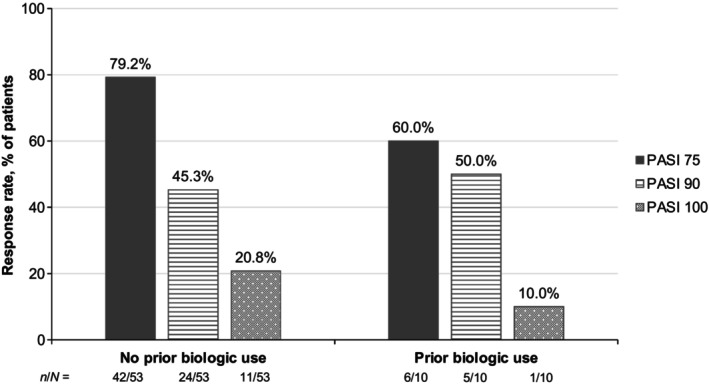
PASI 75/90/100 at week 16 in patients with PP by prior biologic use (as observed). PASI 75/90/100, ≥75%/≥90%/100% reduction from baseline in the Psoriasis Area and Severity Index; PP, plaque psoriasis.

At week 16, 76.2% of patients achieved PASI 75 and 82.5% achieved sPGA 0/1 (Figure 2), with improvements seen as early as week 4. PASI 75 and sPGA 0/1 results were overall comparable at week 16 regardless of baseline body mass index (BMI; 79.4% and 88.2% for BMI <25 kg/m^2^, respectively; 72.4% and 75.9% for BMI ≥25 kg/m^2^) (Figure 3). A numerically higher proportion of patients without prior biologic use achieved PASI 75 and PASI 100 by week 16 (79.2% and 20.8%) compared with those who had been treated with biologics in the past (60.0% and 10.0%) (Figure 4), but achievement of PASI 90 was similar between the two groups (45.3% and 50.0%, respectively).

**Figure 5:**
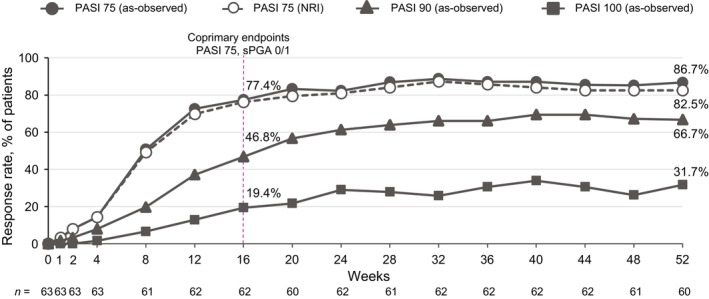
PASI 75/90/100 in patients with PP through week 52. PASI 75 data are presented as both NRI and as observed. PASI 90 and 100 data presented as observed. NRI, nonresponder imputation; PASI 75/90/100, ≥75%/≥90%/100% reduction from baseline in the Psoriasis Area and Severity Index; PP, plaque psoriasis; sPGA 0/1, static Physician's Global Assessment score of 0 (clear) or 1 (almost clear) with at least a two‐point improvement from baseline.

**Figure 6:**
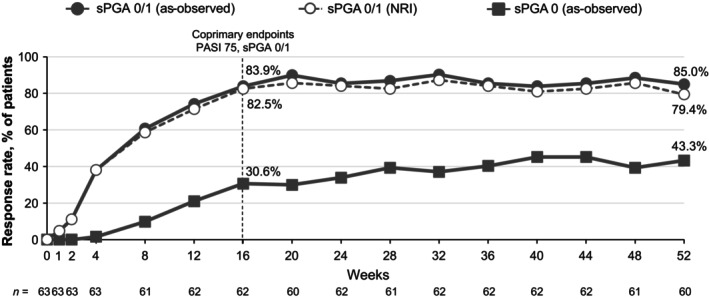
sPGA 0/1 and sPGA 0 in patients with PP. sPGA 0/1 presented as NRI and as observed. sPGA 0 presented as observed. NRI, nonresponder imputation; PASI 75, ≥75% reduction from baseline in the Psoriasis Area and Severity Index; PP, plaque psoriasis; sPGA 0/1, static Physician's Global Assessment score of 0 (clear) or 1 (almost clear) with at least a two‐point improvement from baseline.

PASI 75, PASI 90, and PASI 100 rates continued to improve through week 52 (86.7%, 66.7%, and 31.7%, respectively, as observed) (Figure 5). sPGA 0/1 and sPGA 0 rates showed a similar pattern (week 52: 85.0% and 43.3%, respectively) (Figure 6). A high percentage of patients with moderate to severe scalp involvement (ss‐PGA scores ≥3) at baseline (*n* = 35) achieved ss‐PGA 0/1 by week 16 (91.2%) and week 52 (87.5%), with a mean decrease of 89.4% from baseline in PSSI at week 52 (Table 3). In the limited number of patients with moderate to severe fingernail or palmoplantar involvement at baseline (*n* = 10 and *n* = 4, respectively), PGA‐F 0/1 and pp‐PGA 0/1 achievement was 20% and 75% at week 16, and was improved or was maintained from weeks 16 to 52 (40% and 75%, respectively), as were percentage changes from baseline in mNAPSI and pp‐PASI scores at week 16 (−15.9% and −85.1%, respectively) and week 52 (−44.2% and −95.3%, respectively).

**Table 3:** Scalp, fingernail, and palmoplantar results in patients with plaque psoriasis (as observed).

Measures	Week 16	Week 24	Week 52
Scalp‐related^a^			
ss‐PGA 0/1, *n* (%)	31 (91.2)	28 (82.4)	28 (87.5)
PSSI, mean change from baseline (SD)^b^	−30.2 (15.7)	−29.0 (16.9)	−28.7 (14.8)
PSSI, mean % change from baseline (SD)	−92.6 (15.7)	−88.1 (25.4)	−89.4 (15.5)
Fingernail related^c^			
PGA‐F, *n* (%)	2 (20.0)	2 (20.0)	4 (40.0)
mNAPSI, mean change from baseline (SD)^d^	−9.4 (10.7)	−12.1 (16.3)	−15.4 (12.4)
mNAPSI, mean % change from baseline (SD)	−15.9 (50.5)	−20.3 (97.5)	−44.2 (55.8)
Palmoplantar‐related^e^			
pp‐PGA 0/1, *n* (%)	3 (75.0)	4 (100)	3 (75.0)
pp‐PASI, mean change from baseline (SD)^f^	−19.9 (22.8)	−24.0 (22.2)	−22.7 (22.1)
pp‐PASI, mean % change from baseline (SD)	−85.1 (29.9)	−100 (0.0)	−95.3 (9.4)

Abbreviations: mNAPSI, modified Nail Psoriasis Severity Index; PGA‐F, Physician's Global Assessment–Fingernail; pp‐PASI, palmoplantar Psoriasis Area and Severity Index; pp‐PGA, palmoplantar Physician's Global Assessment; PSSI, Psoriasis Scalp Severity Index; SD, standard deviation; ss‐PGA, scalp‐specific Physician's Global Assessment.

^a^ Patients in this group all had baseline ss‐PGA scores ≥3 (*n* = 35).

^b^ Baseline value = 32.4.

^c^ Patients in this group all had baseline PGA‐F scores ≥3 (*n* = 10).

^d^ Baseline value = 29.7.

^e^ Patients in this group all had baseline pp‐PGA scores ≥3 (*n* = 4).

^f^ Baseline value = 24.0.

#### 3.2.2 Generalized pustular and erythrodermic psoriasis

PASI 75, PASI 90, PASI 100, sPGA 0/1, and sPGA 0 rates were improved at week 16 and generally maintained for patients with GPP and EP from week 16 to week 52 (Table 4). Of the three patients with GPP, two presented with moderate to severe scalp involvement at baseline; no patients achieved ss‐PGA 0/1 at week 16, and one achieved ss‐PGA 0/1 by week 52. No patients with GPP presented with moderate to severe fingernail or palmoplantar involvement at baseline. Of the eight patients with EP, five presented with moderate to severe scalp involvement, four with moderate to severe fingernail involvement, and one with moderate to severe palmoplantar involvement at baseline. At week 16, three of five patients with moderate to severe scalp involvement achieved ss‐PGA 0/1, zero of four with moderate to severe fingernail involvement achieved PGA‐F 0/1, and zero of one with moderate to severe palmoplantar involvement achieved pp‐PGA 0/1; at week 52, three of five patients achieved ss‐PGA 0/1, one of four patients achieved PGA‐F 0/1, and zero of one patient achieved pp‐PGA 0/1 (Table 5). Percentage changes from baseline in PSSI, mNAPSI, and pp‐PASI in GPP and EP patients are also presented in Table 5.

**Table 4:** PASI and sPGA responses over 52 weeks in patients with generalized pustular psoriasis or erythrodermic psoriasis.

Type of psoriasis	Week 16					Week 52				
	PASI 75, %	sPGA 0/1, %	PASI 90, %	PASI 100, %	sPGA 0, %	PASI 75, %	sPGA 0/1, %	PASI 90, %	PASI 100, %	sPGA 0, %
Generalized pustular (*n* = 3)	66.7	0.0	33.3	33.3	33.3	66.7	33.3	33.3	33.3	33.3
Erythrodermic (*n* = 8)	37.5	50.0	12.5	0.0	0.0	37.5	50.0	12.5	12.5	12.5

Abbreviations: PASI, Psoriasis Area and Severity Index; PASI 75/90/100, ≥75%/≥90%/100% reduction from baseline in Psoriasis Area and Severity Index; sPGA 0/1, static Physician's Global Assessment score of 0 (clear) or 1 (almost clear) with at least a two‐point improvement from baseline.

**Table 5:** Scalp, fingernail, and palmoplantar results in patients with generalized pustular psoriasis or erythrodermic psoriasis (as observed).

Outcomes	Week 16	Week 24	Week 52
Generalized pustular psoriasis^a^			
Scalp‐related outcomes (*n* = 2)^b^			
ss‐PGA 0/1, *n* (%)	0.0 (0.0)	0.0 (0.0)	1 (50.0)
PSSI, mean change from baseline (SD)	−20.5 (29.0)	−27.0 (19.8)	−28.5 (6.4)
PSSI, mean % change from baseline (SD)	−45.6 (64.4)	−68.8 (31.6)	−79.5 (8.8)
Erythrodermic psoriasis			
Scalp‐related outcomes (*n* = 5)^b^			
ss‐PGA 0/1, *n* (%)	3 (75.0)	2 (50.0)	3 (75.0)
PSSI, mean change from baseline (SD)	−30.0 (18.2)	−34.3 (19.4)	−39.8 (16.6)
PSSI, mean % change from baseline (SD)	−66.3 (32.9)	−74.2 (32.2)	−87.3 (15.0)
Fingernail‐related outcomes (*n* = 4)^c^			
PGA‐F, *n* (%)	0.0 (0.0)	0.0 (0.0)	1 (50.0)
mNAPSI, mean change from baseline	−10.0 (3.6)	−13.7 (3.1)	−20.0 (1.4)
mNAPSI, mean % change from baseline (SD)	−24.9 (12.8)	−34.0 (12.0)	−61.3 (19.5)
Palmoplantar‐related outcomes (*n* = 1)^d^			
pp‐PGA 0/1, *n* (%)	0.0 (0.0)	1 (100)	0.0 (0.0)
pp‐PASI, mean change from baseline	−15.6 (−)	−19.2 (−)	−3.6 (−)
pp‐PASI, mean % change from baseline (SD)	−72.2 (−)	−88.9 (−)	−16.7 (−)

Abbreviations: mNAPSI, modified Nail Psoriasis Severity Index; PGA‐F, Physician's Global Assessment–Fingernail; pp‐PASI, palmoplantar Psoriasis Area and Severity Index; pp‐PGA, palmoplantar PGA; PSSI, Psoriasis Scalp Severity Index; SD, standard deviation; ss‐PGA, scalp‐specific Physician's Global Assessment.

^a^ There were no patients with generalized pustular psoriasis with fingernail‐ or palmoplantar‐related symptoms.

^b^ Patients in this group all had baseline ss‐PGA scores ≥3.

^c^ Patients in this group all had baseline PGA‐F scores ≥3.

^d^ Patients in this group all had baseline pp‐PGA scores ≥3.

### 3.3 Safety

Safety outcomes are summarized in Table 6. Over 52 weeks, total exposure in patient‐years (PY) was 70.4 (PP, 60.6 PY; GPP, 3.0 PY; EP, 6.8 PY). The most common AEs (occurring in ≥5% of patients with PP) were nasopharyngitis, acne, periodontitis, and upper respiratory tract infection. The incidence of SAEs was low (7.2/100 PY) and did not affect any one organ system in particular. SAEs included T‐cell variant Hodgkin's disease in one EP patient, and asthma, normal pressure hydrocephalus, COVID‐19, and pneumonia in four PP patients. The pneumonia was determined to be community acquired and was treated with standard therapy. Two patients with PP discontinued treatment due to psoriasis aggravation and a decreased neutrophil count; two patients with EP discontinued treatment due to a photosensitivity reaction and Hodgkin's lymphoma reported above. There were no deaths during the study.

**Table 6:** Safety summary, weeks 0–52.

AE category	Plaque psoriasis (*n* = 63)		Generalized pustular psoriasis (*n* = 3)		Erythrodermic psoriasis (*n* = 8)	
	*n* (%)	EAIR/100 PY	*n* (%)	EAIR/100 PY	*n* (%)	EAIR/100 PY
AEs^a^	47 (74.6)	172.6	3 (100)	21915.0^b^	7 (87.5)	279.7
Deaths	0	0	0	0	0	0
Serious AEs^a^	4 (6.3)	6.7	0	0	1 (12.5)	14.6
AEs leading to discontinuation^c^	2 (3.2)	3.3	0	0	2 (25.0)	29.8
AEs occurring ≥5% in PP cohort^d^						
Nasopharyngitis	20 (31.7)	41.4	0	0	3 (37.5)	54.3
Acne	5 (7.9)	8.8	1 (33.3)	47.3	1 (12.5)	16.8
Periodontitis	4 (6.3)	6.8	0	0	0	0
Upper respiratory tract infection	4 (6.3)	6.8	0	0	0	0

Abbreviations: AE, adverse event; EAIR, exposure‐adjusted incidence rate; PP, patients with plaque; PY, person‐years.

^a^ AEs and serious AEs occurring within 30 days after the last dose were recorded.

^b^ There was a small number of patients in this group.

^c^ Includes psoriasis aggravation (*n* = 1) and decreased neutrophil count (*n* = 1) in the plaque psoriasis cohort, and photosensitivity reaction (*n* = 1) and Hodgkin's disease (*n* = 1) in the erythrodermic psoriasis cohort.

^d^ In addition, pyrexia was detected in 3 (4.1%) patients, including 1 (1.6%) patient with plaque psoriasis and 2 (66.7%) patients with generalized pustular psoriasis.

The incidence of AEs of interest was also low (Table 7). Acne and folliculitis events (9.5% and 1.6%) were mild or moderate in severity, none was serious, and none led to treatment discontinuation. These events typically occurred on the face, chest, and back, with a median time to onset of 15.5 days and a median duration of 75.5 days. Six of eight patients (75%) with these skin lesions required treatment with topical agents, including topical and/or oral antibiotics.

**Table 7:** AEs of interest, weeks 0–52.

AEs of interest preferred term	Plaque psoriasis (*n* = 63)		Generalized pustular psoriasis (*n* = 3)		Erythrodermic psoriasis (*n* = 8)	
	*n* (%)	EAIR/100 PY	*n* (%)	EAIR/100 PY	*n* (%)	EAIR/100 PY
Skin events						
Acne	5 (7.9)	8.8	1 (33.3)	47.3	1 (12.5)	16.8
Folliculitis	1 (1.6)	1.6	0	0	0	0
Infection events						
Herpes zoster	1 (1.6)	1.6	0	0	0	0
Malignancy events						
Hodgkin's lymphoma	0	0	0	0	1 (12.5)^a^	14.6
MACE	0	0	0	0	0	0
VTE	0	0	0	0	0	0

Abbreviations: AEs, adverse events; EAIR, exposure‐adjusted incidence rate; MACE, major adverse cardiovascular event; PY, person‐years; VTE, venous thromboembolism.

^a^ This was the only case of Hodgkin's disease that occurred during the study.

The single herpes zoster event in a patient with PP was localized, was not serious, did not result in deucravacitinib discontinuation, was not considered treatment‐related, and resolved with antiviral treatment. No tuberculosis, influenza, or opportunistic infections occurred. No major adverse cardiovascular events (MACE), venous thromboembolism (VTE) or peripheral arterial events, or suicidal ideation or behavioral events were reported.

One malignancy, Hodgkin's lymphoma (mentioned above), was reported as an SAE in a patient with EP that led to deucravacitinib discontinuation. This was a 46‐year‐old male with no previous exposure to phototherapy, conventional systemic or biologic therapy, and no family history of malignancy. He had elevated B‐cell counts at baseline and Epstein–Barr virus DNA detectable at week 1 (Table 8). The AE was observed on day 249 and diagnosis was confirmed on lymph node biopsy on day 261 showing T‐cell variant Hodgkin's lymphoma. This event was not considered treatment‐related by the investigator. The patient received appropriate chemotherapy and is now in remission.

**Table 8:** Laboratory parameters for an erythrodermic psoriasis patient with Hodgkin's lymphoma.

Time point	Cell type						Date	EBV serum marker			
	B cells	Monocyte	NK cells	T cells (CD3+)	Cytotoxic T cells (CD8)	Helper T cells (CD4)		DNA	IgM		
Week 0	1385 (H)	398	204	1394	213	1173	02 Sep 2019	<200	87.5 (H)	VCA ≥750 (H)	EBNA
								W1 257	W1 < 36		57.0 (H)
								W2 518	W2 < 36		53.7 (H)
								W4 211	W4 < 36		56.4 (H)
Week 8	1231 (H)	405	171	1163	179	977	25 Oct 2019	<200	<36	>750 (H)	59.4 (H)
Week 16	1038 (H)	425	203	1254	193	1038	23 Dec 2019	<200	<36	>750 (H)	56.2 (H)
								W24 261	W24 < 36		51.5 (H)
Week 36	584	469	153	415 (L)	112 (L)	297 (L)	12 May 2020	2603	<36	>750 (H)	46.1 (H)
Early termination visit (week ≈40)	468	413	91 (L)	325 (L)	71 (L)	251 (L)	08 Jun 2020	1220	<36	>750 (H)	49.9 (H)
Normal	107–698	NA	95–640	603–2990	125–1312	441–2156					

Abbreviations: EBNA, Epstein–Barr nuclear antigen; EBV, Epstein–Barr virus; H, high; Ig, immunoglobulin; NA, not applicable; NK, natural killer; VCA, viral capsid antigen; W, week.

### 3.4 Laboratory parameters

**Figure 7:**
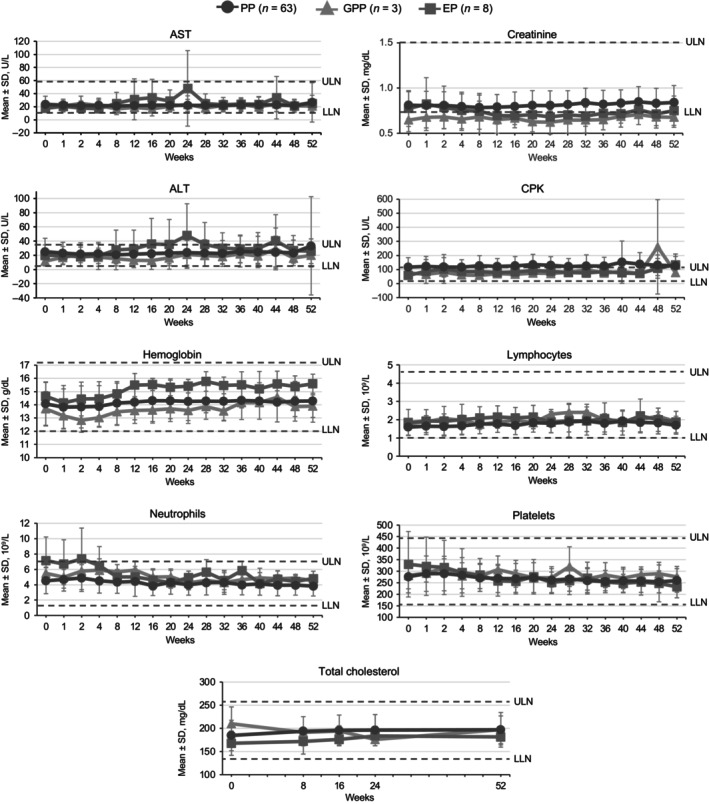
Hematologic, lipid, and laboratory parameters. ALT, alanine aminotransferase; AST, aspartate aminotransferase; CPK, creatine phosphokinase; EP, erythrodermic psoriasis; GPP, generalized pustular psoriasis; LLN, lower limit of normal; PP, plaque psoriasis; SD, standard deviation; ULN, upper limit of normal.

In general, hematologic, lipid, and chemistry parameters were stable over time (Figure 7). One patient with PP with a grade 0 neutrophil count at baseline experienced a transient grade 3 decrease in neutrophil count without significant medical events at week 40 and discontinued treatment. This was considered related to study treatment by the investigator. Grade 3 elevations in aspartate aminotransferase (AST) or alanine aminotransferase (ALT) occurred in one patient with PP, simultaneous with a diagnosis with a nonserious AE of infectious mononucleosis. This patient enrolled in the POETYK LTE study and AST/ALT levels recovered to within normal limits by week 4 of the LTE. Grade 1–2 elevations in AST/ALT occurred in three patients with EP at week 24. Of these, one patient had a reported history of drug‐induced liver injury and a moderate severity AE of alcoholic liver disease leading to treatment interruption and two had histories of hepatic steatosis and type 2 diabetes mellitus. AST/ALT elevations were transient and generally returned to baseline for all three patients by week 36. The majority of patients had creatine phosphokinase (CPK) values in the normal range. One PP patient experienced a grade 2 elevation of ≥2.5× the upper limit of normal at week 40 with no precipitating factors identified. The CPK elevation resolved without treatment after 29 days.

## 4 DISCUSSION

Deucravacitinib was efficacious and well tolerated in Japanese patients with moderate to severe PP, and in a limited number of patients with GPP or EP, based on the responses of the coprimary endpoints of PASI 75 and sPGA 0/1 at week 16. Deucravacitinib elicited improvements as early as week 4, with increased PASI 75 and sPGA 0/1 responses over time. Improvements were seen regardless of baseline BMI or prior treatment status. Deucravacitinib also led to improvements on multiple secondary endpoints, and these responses were maintained over 52 weeks.

Deucravacitinib was generally safe and well tolerated, with nasopharyngitis and acne being the most frequent AEs. SAEs, including serious infections, occurred at a low incidence and did not affect any organ system in particular. No MACE, VTE, tuberculosis, opportunistic infections, or suicidal ideation or behavioral events were reported, in contrast to other agents, such as JAK 1/2/3 inhibitors, which have been associated with increased risks of these cardiovascular events and serious infections.^24^, ^25^ The one case of herpes zoster was localized, not serious, and not considered related to treatment.

Malignancy was considered an AE of interest due to the increased risk of malignancies inherent in patients with psoriasis, as well as the potential increase in risk associated with systemic, biologic treatments.^26^, ^27^ One case of T‐cell variant Hodgkin's lymphoma in a patient with EP was reported and considered by the investigator to be not related to deucravacitinib treatment. Based on the laboratory results of elevated B cells and the positive result for Epstein–Barr virus at the beginning of the trial, a known risk factor for Hodgkin's lymphoma,^28^ the malignancy may have been a preexisting condition in the patient when he entered the trial.

The efficacy and safety results in patients with PP in this trial were consistent with those from Japanese patients (*n* = 66) included in the randomized, double‐blind, global phase 3 POETYK PSO‐1 trial.^22^ In POETYK PSO‐1, the week 16 PASI 75 response rate was 78.1% and the sPGA 0/1 rate was 75.0% in the Japanese subpopulation, compared with 76.2% and 82.5%, respectively, in the population with PP in the POETYK PSO‐4 trial. The week 16 PASI 75 and sPGA 0/1 responses in the current study are numerically higher than those seen in the overall populations of PSO‐1 (PASI 75, 58.4%; sPGA 0/1, 53.6%) and PSO‐2 (PASI 75, 53.0%; sPGA 0/1, 49.5%).^20^, ^21^ A possible reason for the higher response rates is the lower body weight seen in Japanese patients.^29^, ^30^

One limitation of the trial is the small number of patients with GPP (*n* = 3) and EP (*n* = 8) enrolled, which makes it difficult to draw clear conclusions about the full effect of deucravacitinib in patients with these types of psoriasis in Japan. Other limitations include the predominance of male (77%) versus female (23%) patients, although this is consistent with what is known of psoriasis prevalence and severity in Japan.^4^ Finally, longer‐term, real‐world evidence is needed to understand the efficacy and safety of deucravacitinib in psoriasis patients over time. The ongoing open‐label LTE trial should provide these long‐term data.

In conclusion, deucravacitinib was well tolerated and efficacious over 52 weeks in patients with PP and in a limited number of patients with GPP and EP in this regional POETYK PSO‐4 study conducted in Japan. These findings, in conjunction with the analysis of Japanese patients in the global POETYK PSO‐1 trial, support the robust efficacy and safety profile of deucravacitinib in Japanese patients with moderate to severe psoriasis.

## CONFLICT OF INTEREST STATEMENT

S.I. has received grants and personal fees from AbbVie, Eisai, Janssen, Kyowa Kirin, Leo Pharma, Maruho, Sun Pharma, Taiho Yakuhin, Tanabe Mitsubishi, and Torii Yakuhin, and has received personal fees from Amgen (Celgene), Boehringer Ingelheim, Bristol Myers Squibb, Daiichi Sankyo, Eli Lilly, GlaxoSmithKline, Novartis, and UCB. Y.O. has received research grants from AbbVie, Eisai, Maruho, Shiseido, Sun Pharma, and Torii Pharmaceutical; has received honoraria from AbbVie, Amgen, Boehringer Ingelheim, Bristol Myers Squibb, Eisai, Eli Lilly, Janssen Pharma, Jimro, Kyowa Kirin, Leo Pharma, Maruho, Novartis Pharma, Pfizer, Sanofi, Sun Pharma, Taiho Pharmaceutical, Tanabe‐Mitsubishi, Torii Pharmaceutical, and UCB; and has conducted clinical trials for AbbVie, Amgen, Boehringer Ingelheim, Bristol Myers Squibb, Celgene, Eli Lilly, Janssen, Leo Pharma, Maruho, Pfizer, Sun Pharma, and UCB. Y.T. has received research grants from AbbVie, Amgen, Boehringer Ingelheim, Bristol Myers Squibb, Eisai, Eli Lilly, Jimro, Kyowa Kirin, Leo Pharma, Maruho, Sun Pharma, Taiho Pharmaceutical, Tanabe‐Mitsubishi, Torii Pharmaceutical, and UCB; has received honoraria from AbbVie, Amgen, Boehringer Ingelheim, Bristol Myers Squibb, Eisai, Eli Lilly, Janssen, Jimro, Kyowa Kirin, Leo Pharma, Maruho, Novartis, Pfizer, Sun Pharma, Taiho Pharmaceutical, Tanabe‐Mitsubishi, Torii Pharmaceutical, and UCB; and has received consulting fees from AbbVie, Boehringer Ingelheim, Bristol Myers Squibb, Eli Lilly, Janssen, Maruho, Novartis, Taiho Pharmaceutical, and UCB. M.O. has received honoraria and/or research grants from AbbVie, Amgen, Boehringer Ingelheim, Bristol Myers Squibb, Celgene, Eisai, Eli Lilly, Janssen, Kyowa Kirin, Leo Pharma, Maruho, Mitsubishi Tanabe Pharma, Nichi‐Iko, Nippon Kayaku, Novartis, Pfizer, Sanofi, Sun Pharma, Taiho Pharmaceutical, Torii Pharmaceutical, and UCB. E.C., A.N., Y.S., and S.B. are employees of and shareholders in Bristol Myers Squibb. A.M. has received honoraria as meeting chair/lecturer for AbbVie, AYUMI Pharmaceutical, Boehringer Ingelheim Japan, Celgene K.K., Eisai, Eli Lilly Japan K.K., Inforward, Janssen Pharmaceutical K.K., Kyowa Kirin, Maruho Co., Mitsubishi Tanabe Pharma, Nippon Kayaku, Novartis Pharma K.K., Taiho Pharmaceutical, Torii Pharmaceutical, and Ushio; has received funding from AbbVie GK, Eisai, Eli Lilly Japan K.K., Kyowa Kirin, Leo Pharma K.K., Maruho, Mitsubishi Tanabe Pharma, Novartis Pharma K.K., Taiho Pharmaceutical, and Torii Pharmaceutical; and has received consulting fees from AbbVie GK, Boehringer Ingelheim Japan, Bristol Myers Squibb, Celgene K.K., Eli Lilly Japan K.K., GlaxoSmithKline K.K., Janssen Pharmaceutical K.K., Kyowa Kirin, Maruho, Mitsubishi Tanabe Pharma, Nichi‐Iko Pharmaceutical, Nippon Kayaku, Novartis Pharma K.K., Pfizer Japan, Sun Pharma, Torii Pharmaceutical, and UCB Japan. Shinichi Imafuku and Yayoi Tada are Editorial Board members of *Journal of Dermatology* and co‐authors of this article. To minimize bias, they were excluded from all editorial decision‐making related to the acceptance of this article for publication.
